# Wax Deposition Inhibition Performance of a PAO/Pour Point Depressant/SiO_2_-IL Ternary System in High-Wax-Content Model Oil

**DOI:** 10.3390/molecules31203667

**Published:** 2026-10-14

**Authors:** Penggang Zhang, Mingji Huang, Jinsong Liu, Banghui Deng, Donglei Yang, Junkai Zhu, Ying Guo

**Affiliations:** Tarim Oilfield Company, PetroChina, Korla 841000, China; zhangpenggang-tlm@petrochina.com.cn (P.Z.); huangmingji-tlm@petrochina.com.cn (M.H.); ljs-tlm@petrochina.com.cn (J.L.); dengyufa-tlm@petrochina.com.cn (B.D.); zhujunkai-tlm@petrochina.com.cn (J.Z.); guoying-tlm@petrochina.com.cn (Y.G.)

**Keywords:** PAO drag-reducing agent, pour point depressant, SiO_2_-IL, wax deposition inhibition, ternary formulation

## Abstract

Wax deposition in high-wax-content oils can impair low-temperature transport, while single flow improvers often provide limited control over the overall deposition process. To improve wax deposition inhibition through functional complementarity, a PAO/pour point depressant (PPD)/SiO_2_-IL ternary formulation was developed. Alkyl-imidazolium-functionalized SiO_2_ materials with C2, C4, and C6 chains were prepared and characterized by FT-IR, TGA, and SEM. Wax deposition was evaluated using a static cold-finger method at a bulk-oil temperature of 55 °C and a cold-finger temperature of 25 °C. A model oil containing 20 wt% paraffin and a deposition time of 2 h was selected for formulation evaluation. The binary formulation containing 40 ppm PAO and 100 ppm JN-3 achieved a wax deposition inhibition efficiency of 67.53%. Among the SiO_2_-IL materials, Sil@HIL (C6) showed comparatively high performance, with its highest inhibition at 200 ppm within the tested dosage range. The resulting ternary formulation achieved 77.49% inhibition, which is 9.96 percentage points above the binary formulation. These findings indicate that incorporating Sil@HIL into the PAO/JN-3 formulation further improves wax deposition inhibition under the investigated static conditions.

## 1. Introduction

As oil and gas development continues, crude oils with high wax contents, high pour points and poor low-temperature flowability have become increasingly important production targets [1]. More than 80% of the crude oil produced in China is classified as waxy crude [2], and the wax content of typical Daqing crude oil can reach approximately 26 wt% [3]. When the oil temperature falls below the wax appearance temperature (WAT), dissolved wax gradually precipitates and undergoes nucleation, growth and aggregation, ultimately forming a wax-crystal network. Some waxy crude oils gel appreciably even when the precipitated wax fraction is only about 2–3 wt% [4]. During pipeline transport, wax components also migrate toward the cooler pipe wall under the combined action of temperature and concentration gradients and form a deposit, reducing the flow area and increasing frictional resistance and energy consumption. In severe cases, this can impede restart after shutdown or even block the pipeline [5]. Pipeline transport of high-wax-content crude oil therefore requires simultaneous hydraulic drag reduction and control of low-temperature wax deposition.

Ultra-high-molecular-weight poly-α-olefin (PAO) drag-reducing agents can substantially reduce pipeline friction by modifying turbulent structures and thereby decreasing turbulent energy dissipation [6]. Hassanean et al. [6] reported that 60 ppm of a PAO drag-reducing agent reduced the pressure loss by approximately 36% and increased the transport capacity of a waxy crude-oil pipeline by approximately 38%. However, the primary function of PAO remains flow regulation, and its effects on wax-crystal formation and deposition depend strongly on concentration and operating conditions. Lv et al. [7] found that 5 mg/kg of drag-reducing agent reduced the mass of wax deposit by no more than 15.66% in a high-wax-content oil, whereas stronger polymer adhesion at higher dosages and under specific flow conditions could even increase wax deposition. Thus, although PAO provides effective drag reduction, its capacity to regulate wax deposition in high-wax-content oils is limited and strongly dependent on concentration and operating conditions.

Polymeric pour point depressants (PPDs) primarily alter wax-crystal size, morphology and aggregation through mechanisms such as co-crystallization, adsorption and steric hindrance, thereby improving the low-temperature flowability of waxy crude oils [8]. Their effectiveness, however, is highly crude-oil-specific and is strongly influenced by oil composition and thermal history [9]; the heat-treatment temperature is particularly important [10]. Yang et al. [10] found that 50 ppm EVA had a weak effect when the heat-treatment temperature was below 50 °C, but its performance improved markedly at 50–60 °C, indicating that suitable high-temperature treatment is important for fully activating a PPD. Lv et al. [11] noted that PAO chains readily undergo scission under the high shear generated by pumps, thereby reducing drag-reduction efficiency. In practical pipelines, PAO is therefore generally injected away from high-shear equipment such as pumps. Field studies have further shown that PPD injection should account for the WAT of the crude oil and should generally be completed, together with mixing, while the fluid temperature remains above the WAT and before extensive wax crystallization [12]. PPD injection is therefore constrained mainly by oil temperature and thermal history, whereas PAO injection must account primarily for mechanical shear. The optimal injection locations and process windows of the two additives do not completely overlap, increasing the operational complexity of their combined use in pipelines.

These constraints have stimulated interest in combined flow-improvement formulations that provide both drag reduction and pour-point depression. Banerjee et al. [13] reported that a polymeric PPD reduced wax deposition while providing approximately 15% drag reduction, indicating that a single additive can, to some extent, combine wax inhibition with drag reduction. However, the PPD dosage used in that study reached 1000 ppm, leaving scope to optimize both functionality and dosage. Cao et al. [14] found that neither a drag-reducing agent nor a PPD altered the WAT or appreciably reduced wax deposition in the pipeline system they examined. These observations indicate that existing single additives have practical performance boundaries and that the design of multifunctional formulations should consider functional complementarity among components.

The incorporation of nanomaterials has recently introduced an additional means of regulating the behavior of conventional polymeric flow improvers. Nanoparticles have high specific surface areas and abundant interfacial interaction sites and can participate in wax crystallization through heterogeneous nucleation and interfacial interactions [15]. Introducing a nanocomponent with an independent capacity to regulate wax crystals into a PAO/PPD system may therefore extend the functional range of conventional polymer formulations. For example, an EVA-modified nano-SiO_2_ composite PPD produced a markedly greater pour-point reduction than a simple physical mixture of the same components [16], indicating that appropriately constructed nanoscale interfaces can strengthen polymer-mediated regulation of wax crystallization. Applying the same concept to a PAO/PPD system may therefore overcome some of the performance limits of a conventional binary formulation.

Ionic liquids (ILs) have increasingly been used to regulate the low-temperature behavior of waxy crude oils because their structures can be designed, their polarity can be tuned, and they can provide strong interfacial interactions. Shao et al. [17] studied imidazolium ionic-liquid surfactants with different alkyl-chain lengths and found that chain length substantially affected oil-water interfacial activity and emulsification performance. Increasing the alkyl-chain length from C12 to C16 enhanced interfacial activity, showing that the alkyl-chain structure of imidazolium ILs is an important determinant of their interfacial behavior. Hazrati et al. [18] found that a C14 long-chain imidazolium IL performed better than its C12 analogue. At 2000 ppm, [C14 mim][NTf2] reduced the pour point of crude oil by approximately 12 °C and markedly lowered its low-temperature viscosity; differential scanning calorimetry and polarized light microscopy indicated that the long-chain IL altered wax-crystal formation and aggregation. Sharma et al. [19] further developed an imidazolium IL/graphene oxide nanocomposite that reduced the pour point of crude oil from 39 °C to approximately 9 °C. These results indicate that ILs can both improve the low-temperature flowability of waxy crude oils directly and serve as components of nanomaterial-based flow-improvement formulations.

Nano-SiO_2_ can likewise affect paraffin nucleation, growth and aggregation. Wang et al. [20] found that SiO_2_ nanoparticles altered paraffin crystallization and that organic-chain surface modification further enhanced their control over nucleation and promoted the formation of smaller wax crystals. Lei et al. [21] introduced an imidazolium IL structure onto the surface of SiO_2_ to construct a SiO_2_/IL composite, showing that imidazolium ILs can serve as organic functional groups on SiO2 surfaces, which provides materials for designing functional SiO_2_-IL nanoparticles.

Building on these studies, we functionalized nano-SiO_2_ with imidazolium ILs of different alkyl-chain lengths to prepare functional SiO_2_-IL nanocomponents. These materials were incorporated into a PAO/PPD system to construct a PAO/PPD/SiO_2_-IL ternary formulation. Cold-finger wax deposition tests were first used to identify a suitable deposition time and model-oil paraffin content and to screen PAO/PPD binary formulations. We then examined how the surface alkyl structure and dosage of SiO_2_-IL affected wax deposition and evaluated both the wax deposition inhibition performance of the ternary formulation and its functional complementarity relative to individual additives and binary formulations. FT-IR, TGA and SEM were used to characterize the chemical structure, surface functionalization and microscopic morphology of SiO_2_-IL. Polarized light microscopy was used to examine wax-crystal size, shape and aggregation under different additive conditions. The combined effects of PAO, the PPD and SiO_2_-IL were analyzed in terms of wax migration and deposition regulation, control of wax-crystal nucleation and growth, and nanoscale interfacial interactions.

## 2. Results and Discussion

### 2.1. Characterization Results

#### 2.1.1. FT-IR

Figure 1 shows that unmodified SiO_2_ exhibited a broad absorption band at approximately 3440 cm^−1^, mainly attributable to O-H stretching vibrations in surface Si-OH groups and adsorbed water. The strong absorption band at approximately 1080 cm^−1^ was assigned to Si-O-Si stretching and is characteristic of the SiO_2_ framework [22].

Compared with unmodified SiO_2_, Sil@EIL, Sil@BIL and Sil@HIL retained the characteristic Si-O-Si framework band but also exhibited aliphatic C-H stretching absorption near 2925 cm^−1^ and characteristic absorption associated with the imidazole-ring framework near 1570 cm^−1^. Studies of SiO_2_-supported alkyl-imidazolium ILs have similarly observed alkyl C-H stretching vibrations at approximately 2917–2849 cm^−1^ and detected characteristic imidazole-ring bands [23]. These results indicate that alkyl-imidazolium organic structures were successfully introduced onto the SiO_2_ surface while the principal siloxane framework of SiO_2_ was retained.

Overall, the FT-IR spectra of Sil@EIL, Sil@BIL and Sil@HIL retained the main features of the SiO_2_ siloxane framework while exhibiting characteristic absorption associated with alkyl chains and imidazole structures, indicating that alkyl-imidazolium organic structures were introduced onto the SiO_2_ surface. The similar principal bands of the three samples are consistent with a common SiO_2_ substrate and imidazolium functional structure, differing only in alkyl-chain length.

#### 2.1.2. TGA

As shown in Figure 2, all samples exhibited some mass loss at low temperature, mainly owing to the removal of surface-adsorbed water and small amounts of residual volatile components. At higher temperatures, the mass of unmodified SiO_2_ changed relatively little, whereas Sil@EIL, Sil@BIL and Sil@HIL showed further mass loss in the intermediate- and high-temperature regions. Previous work has attributed the additional mass loss of IL-functionalized SiO_2_ at approximately 200–600 °C mainly to the thermal decomposition of the organic surface groups [23].

At 800 °C, the total mass losses of SiO_2_, Sil@EIL, Sil@BIL and Sil@HIL were approximately 7.97%, 8.84%, 9.16% and 11.07%, respectively. The total mass loss of each modified sample exceeded that of unmodified SiO_2_, indicating the presence of additional organic components after functionalization. Together with the FT-IR results, these data further support the successful introduction of imidazolium organic structures with different alkyl-chain lengths onto the SiO_2_ surface. The residual mass (char yield) at 800 °C decreased in the same order: 92.03% for unmodified SiO_2_, 91.16% for Sil@EIL, 90.84% for Sil@BIL and 88.93% for Sil@HIL. Because the residue is dominated by the thermally stable siliceous framework, the decrease in char yield follows the increase in the amount of organic surface material. The additional mass loss of the functionalized samples occurred mainly between approximately 200 and 600 °C, which is the range in which decomposition of the grafted imidazolium and alkoxysilane-derived organic groups is expected [23]. No pronounced mass loss was observed above 600 °C, indicating that under an inert nitrogen atmosphere, the grafted organic layer decomposes almost completely rather than forming a thermally stable carbonaceous char; the residue of the modified samples therefore consists essentially of the silica framework together with a small amount of carbonized material.

#### 2.1.3. SEM

Sil@EIL, Sil@BIL and Sil@HIL displayed similar particle morphologies at the same SEM magnification, with no pronounced differences among the three samples. Sil@HIL was therefore selected as a representative sample and compared with unmodified SiO_2_ to examine the changes in particle morphology after IL surface functionalization.

Figure 3 shows that the unmodified SiO_2_ particles were generally regular and near-spherical, with relatively smooth surfaces and a comparatively uniform size distribution. After IL modification, the particles generally retained their near-spherical outlines, with no obvious disruption of their overall morphology. Their surfaces, however, became noticeably rougher and displayed localized irregular protrusions.

Similar morphological changes have been reported for IL-functionalized SiO_2_. Chen et al. [24] found that unmodified SiO_2_ particles had relatively smooth, near-spherical surfaces, whereas the introduction of an imidazolium IL increased surface roughness and produced a distinct surface-coating structure. Nguyen et al. [23] likewise found that immobilizing an alkyl-imidazolium IL appreciably altered the surface morphology of SiO_2_ and produced a rougher surface.

Together with the FT-IR and TGA results, the SEM observations indicate that IL modification did not appreciably disrupt the basic particle morphology of SiO_2_ but did alter its microscopic surface structure and the state of interparticle interactions, providing further morphological support for SiO_2_ surface functionalization.

### 2.2. Effect of Paraffin Content on Wax Deposition

To identify a model-oil composition suitable for the subsequent evaluation of additives and formulations, wax deposition was examined at paraffin mass fractions of 10, 15, 20, 25 and 30 wt% with the PAO dosage fixed at 40 ppm. In accordance with the experimental design, the deposition time was fixed at 2 h during paraffin-content screening, and all other cold-finger conditions were held constant.

The mass of wax deposited on the cold-finger surface increased markedly with paraffin content (Table 1). At paraffin contents of 10 wt%, 15 wt% and 20 wt%, the mean deposited masses were 0.19, 0.32 and 0.51 g, respectively. Increasing the paraffin content to 25 wt% and 30 wt% raised the mean deposited masses to 1.02 and 1.95 g, respectively. Increasing the paraffin content of the model oil therefore increased wax deposition on the low-temperature surface, consistent with previous observations that a higher wax content promotes wax precipitation and deposition in model wax-oil systems [25].

At 10 wt% and 15 wt%, the deposited masses were relatively small and therefore more susceptible to variation caused by liquid entrainment on the cold finger, oil drainage and weighing error, making quantitative comparison among additive formulations difficult. At 20 wt%, the mean deposited mass reached 0.51 g and the three parallel measurements were closely grouped, indicating that this condition produced a deposit with sufficient mass for discrimination and good experimental reproducibility.

At 25 wt% and 30 wt%, both the mean deposited mass and the dispersion among the three parallel measurements increased markedly. As the precipitated wax-phase content increases, the viscosity and non-Newtonian characteristics of waxy oil become more pronounced [26]. Studies of model oils with different wax concentrations have further shown that gelation temperature increases and the wax-crystal network develops with increasing wax content, indicating a stronger propensity for gelation in high-wax-content systems [25]. At high wax contents, wax-crystal polydispersity and interconnection more readily form mechanical gel networks that entrap liquid oil [27]. Deposits weighed at high paraffin contents may therefore contain more liquid oil trapped within the wax-crystal network, increasing experimental variability.

Considering measurability, operability and the reproducibility of parallel experiments, the model oil containing 20 wt% paraffin formed a clear and stable wax deposit. This condition avoided the poor discrimination associated with the low deposited mass at lower paraffin contents while reducing the experimental variability that could arise from stronger gelation and liquid-oil entrapment at higher paraffin contents. A model oil containing 20 wt% paraffin was therefore used in all subsequent experiments on the PAO/PPD and PAO/PPD/SiO_2_-IL formulations.

### 2.3. Effect of Deposition Time on Wax Deposition

Wax deposition was then examined at 0.5, 1, 2, 3, 4 and 6 h, with the PAO dosage fixed at 40 ppm and all other conditions held constant, to select a common deposition time for subsequent evaluation of individual additives and formulations. Because 20 wt% paraffin and 2 h represented the intersection of the two screening experiments, the same three parallel measurements were used for that condition.

Deposition time strongly affected the mass of wax deposited on the cold-finger surface (Table 2). During the first 0.5–2 h, the mean deposited mass increased from 0.25 g to 0.40 g and then to 0.51 g, indicating that this interval was the main growth stage under the experimental conditions.

When the deposition time was extended to 3, 4 and 6 h, the mean deposited masses were 0.48, 0.51 and 0.48 g, respectively, with no continued increase over time. The system therefore gradually entered a relatively stable stage under these conditions. Lee et al. [28] likewise found that a multicomponent wax deposit on a cold surface initially grew with time before gradually approaching a stable thickness. Rotating cold-finger experiments have also shown the mass of wax deposit approaching a quasi-steady state over time [29].

When the oil first contacts the cold finger, pronounced temperature and wax-concentration gradients develop near the cold surface. The lower local temperature induces wax precipitation, and the concentration gradient drives wax toward the surface, gradually forming an initial deposit [30]. As the deposit forms and thickens, its additional thermal resistance increases and impedes heat transfer between the cold surface and the bulk oil phase. This progressively weakens both the temperature gradient near the deposition interface and the driving force for wax migration, lowering the subsequent deposition rate and causing the process to approach a stable state [31].

Considering the variation in deposited mass, experimental reproducibility and testing efficiency, a deposition time of 2 h produced a wax layer that could be weighed accurately and captured the main deposition behavior of the system. Extending the experiment to 6 h did not yield an appreciably larger deposit and instead lengthened the testing period without providing additional discriminatory information. A deposition time of 2 h was therefore used in all subsequent experiments.

### 2.4. Concentration Screening and Performance Evaluation of PAO/PPD Binary Formulations

#### 2.4.1. Effect of PPD Dosage on Wax Deposition by the Binary Formulations

Under the standardized conditions described above, the blank without any flow improver had a mean deposited mass of 0.77 ± 0.04 g (*n* = 3). Wax deposition inhibition efficiencies for all subsequent additive systems were calculated relative to this blank value.

PAO dosage was fixed at 40 ppm, paraffin mass fraction at 20 wt% and deposition time at 2 h. With all other conditions held constant, EVA and JN-3 were each tested at 20, 40, 60, 80 and 100 ppm.

Table 3 and Table 4 and Figure 4 show that the mean deposited mass decreased as the concentration of either EVA or JN-3 increased from 20 ppm to 100 ppm at a fixed PAO dosage of 40 ppm. Within the 20–100 ppm range examined, increasing the PPD dosage therefore improved the inhibition of wax deposition on the cold surface by the PAO/PPD formulations.

For the PAO/EVA formulation, EVA dosages of 20, 40, 60, 80 and 100 ppm yielded mean deposited masses of 0.50, 0.48, 0.47, 0.42 and 0.36 g, respectively. The corresponding wax deposition inhibition efficiency increased from 35.06% to 53.25%. The deposited mass decreased relatively slowly between 20 ppm and 60 ppm, but more markedly at 80 ppm and 100 ppm, indicating a concentration-dependent effect of EVA on wax deposition in the model oil.

The polyethylene segments in EVA resemble paraffin hydrocarbon chains and can participate in wax-crystal formation, whereas the polar vinyl acetate groups can disrupt the regular arrangement of wax molecules and crystal growth, thereby altering wax-crystal morphology and aggregation. Molecular simulations by Dai et al. [32] showed that EVA altered wax-crystal morphology and that its wax-inhibition performance was closely related to the content and spatial distribution of polar vinyl acetate units. The reduction in deposited mass with increasing EVA concentration observed here may therefore reflect the increasing number of EVA molecules participating in wax-crystal regulation and the stronger disruption of wax-crystal growth and aggregation.

For the PAO/JN-3 formulation, increasing JN-3 from 20 ppm to 100 ppm reduced the mean deposited mass from 0.45 g to 0.25 g and increased the wax deposition inhibition efficiency from 41.99% to 67.53%. At the same PPD dosage, the PAO/JN-3 formulation generally produced a lower deposited mass than the PAO/EVA formulation. At 20, 60 and 100 ppm, for example, the wax deposition inhibition efficiencies of PAO/JN-3 were 41.99%, 55.41% and 67.53%, respectively, exceeding the corresponding values of 35.06%, 39.39% and 53.25% for PAO/EVA. At 100 ppm, the 40 ppm PAO/100 ppm JN-3 formulation produced a mean deposited mass of 0.25 g, the lowest value among the binary formulations containing the two candidate PPDs. Under the preparation and experimental conditions used here, the PAO/JN-3 formulation exhibited a higher measured wax deposition inhibition efficiency than the PAO/EVA formulation.

The dissolution and phase behavior of EVA are known to depend strongly on the solvent, and aromatic hydrocarbons such as xylene have been used to prepare homogeneous EVA-based formulations. Ragab et al. [33], for example, used xylene as the medium for preparing an EVA-based PPD. However, xylene cannot be regarded as a completely inert carrier solvent in wax-deposition experiments. Ridzuan and Al-Mahfadi [34] showed that xylene itself could affect wax deposition in crude oil. Because an independent xylene control was not included in the present experiments, the contribution of xylene to the measured performance of the EVA-containing systems cannot be completely separated from that of EVA.

Considering both the mean deposited mass and wax deposition inhibition efficiency, each candidate PPD showed its highest formulation performance at 100 ppm within the 20–100 ppm range examined. The 40 ppm PAO/100 ppm EVA and 40 ppm PAO/100 ppm JN-3 formulations were therefore selected as representative systems for subsequent comparisons between individual additives and formulations. Of these, PAO/JN-3 inhibited wax deposition more effectively than PAO/EVA.

#### 2.4.2. Wax Deposition Inhibition Performance of the PAO/PPD Binary Formulations

100 ppm EVA or 100 ppm JN-3 was combined with 40 ppm PAO and compared with the corresponding individual additives. Wax deposition inhibition efficiency (PIE) was used as the principal metric for assessing the performance of PAO combined with each PPD (Table 5).

The additive systems differed substantially in their ability to inhibit wax deposition on the cold surface of the waxy model oil (Figure 5). When used individually, 40 ppm PAO, 100 ppm EVA and 100 ppm JN-3 yielded PIE values of 33.33%, 24.24% and 50.65%, respectively. JN-3 showed the highest inhibition among the individual additives, followed by PAO, whereas EVA had a relatively low PIE in this model oil. Adams et al. [9] noted that crude oils with similar wax contents can respond differently to the same type of PPD, indicating a strong dependence on the compatibility between the PPD and the specific oil phase.

Combining PAO with PPD increased the PIE above that of the corresponding individual additives. Combining 40 ppm PAO with 100 ppm EVA increased the PIE to 53.25%, 19.92 percentage points above PAO alone and 29.01 percentage points above EVA alone. Combining 40 ppm PAO with 100 ppm JN-3 increased the PIE to 67.53%, 34.20 percentage points above PAO alone and 16.88 percentage points above JN-3 alone. Thus, under the present experimental conditions, combining PAO with PPD inhibited wax deposition more effectively than the corresponding individual additives, with PAO/JN-3 giving the highest performance.

The further increase in PIE indicates that combining PAO with a PPD improved the inhibition of wax deposition under the present experimental conditions. In addition to their conventional role in turbulent drag reduction, drag-reducing agents may also influence wax deposition in waxy systems [35], whereas PPDs act primarily by modifying wax-crystal growth and aggregation. PAO and the PPD may therefore influence wax deposition through different pathways.

The PIE of PAO/JN-3 was 67.53%, exceeding the 53.25% obtained with PAO/EVA. The concentration-dependent comparison in Section 3.4.1 likewise showed that, at the same PPD dosage, PAO/JN-3 generally had a higher PIE than PAO/EVA. Under the preparation and experimental conditions used here, PAO/JN-3 therefore showed higher wax deposition inhibition, and the formulation containing 40 ppm PAO and 100 ppm JN-3 was selected as the binary base for the subsequent experiments.

### 2.5. Effects of SiO_2_-IL Alkyl-Chain Length and Dosage on Wax Deposition by the PAO/JN-3 Formulation

After selecting 40 ppm PAO/100 ppm JN-3 as the base binary formulation, SiO_2_-IL materials with different alkyl-chain lengths were introduced at different dosages to systematically examine how the surface structure and dosage affected wax deposition. Sil@EIL, Sil@BIL and Sil@HIL were each tested at 0, 50, 100, 200, 300 and 500 ppm, with the 0 ppm group representing the PAO/JN-3 binary formulation without SiO_2_-IL. Wax deposition inhibition efficiencies were compared to assess the effects of the SiO_2_-IL surface alkyl-chain structure and dosage on the performance of the PAO/JN-3 formulation.

The extent to which SiO_2_-IL improved the wax deposition performance of PAO/JN-3 differed markedly with alkyl-chain length (Figure 6). Within the C2-C6 range examined, Sil@HIL generally showed greater improvement than Sil@EIL and Sil@BIL and achieved the highest PIE within the investigated concentration range. The SiO_2_-IL materials with different surface structures therefore exhibited different wax deposition inhibition performances in the waxy oil. The alkyl-chain length of imidazolium ILs is known to alter the interfacial structure, surface orientation and interfacial organization. Watanabe et al. [36] found that the alkyl-chain length of the imidazolium cation changed the molecular arrangement and the structure of the interfacial layer. Molecular simulations by Pensado et al. [37] likewise showed that changing the alkyl-chain structure of an imidazolium cation altered surface tension, interfacial order and density distribution.

The three SiO_2_-IL samples differed not only in surface alkyl-chain structure but also in TGA mass loss. Their performance differences therefore cannot be attributed solely to alkyl-chain length and may also be influenced by the amount of organic surface material, particle dispersion and the state of interfacial interactions. At 800 °C, the total mass losses of Sil@EIL, Sil@BIL and Sil@HIL were approximately 8.84%, 9.16% and 11.07%, respectively, all higher than the 7.97% measured for unmodified SiO_2_, and the modified samples also differed from one another. The observed performance differences among the SiO_2_-IL materials are therefore more likely to reflect the combined effects of surface alkyl-chain structure, organic-functional-group loading, dispersion in the oil phase and interfacial interactions. Under the preparation conditions used here and within the C2–C6 range examined, Sil@HIL showed comparatively high wax deposition inhibition.

The effect of the Sil@HIL dosage on the wax deposition inhibition performance of the PAO/JN-3 formulation exhibited a clear concentration-dependent trend. Without SiO_2_-IL, the PAO/JN-3 binary formulation exhibited a PIE of 67.53%. As the Sil@HIL dosage increased to 50, 100 and 200 ppm, the PIE increased to 71.43%, 74.03% and 77.49%, respectively. Further increasing the Sil@HIL dosage to 300 and 500 ppm reduced the PIE to 73.16% and 69.70%, respectively. Thus, within the dosage range investigated, the improvement produced by Sil@HIL first increased and then decreased with increasing dosage. The highest PIE was obtained at 200 ppm, which was 9.96 percentage points higher than that of the PAO/JN-3 binary formulation without SiO_2_-IL.

This rise-then-fall concentration response resembles that reported for other nano-SiO_2_-based wax-inhibition formulations. In a cold-finger study of polymer/SiO_2_-nanoparticle formulations, Elarbe et al. [38] identified an appropriate SiO_2_ dosage beyond which further addition of nanoparticles did not produce additional improvement in wax deposition inhibition. This observation suggests that the performance of nanoparticle-containing formulations does not necessarily increase continuously with nanoparticle dosage. One possible explanation for the decrease in PIE at higher Sil@HIL dosages is that increased interparticle interactions may promote particle aggregation, thereby reducing the effective interfacial area available for interaction with wax components. However, particle aggregation and dispersion behavior were not directly characterized in the present study, and this interpretation therefore requires further experimental verification.

Considering all alkyl structures and dosages, Sil@HIL (C6) showed comparatively high wax deposition inhibition within the C2–C6 range examined, with a selected dosage of 200 ppm. The formulation containing 40 ppm PAO, 100 ppm JN-3 and 200 ppm Sil@HIL was therefore used for the subsequent ternary-formulation evaluation.

### 2.6. Wax Deposition Inhibition Performance of the PAO/JN-3/SiO_2_-IL Ternary Formulation

On the basis of the preceding screening results, the formulation containing 40 ppm PAO, 100 ppm JN-3 and 200 ppm Sil@HIL was selected as the ternary formulation. For comparative evaluation, wax deposition results obtained under the same standardized conditions were compiled for the blank, 40 ppm PAO, 100 ppm JN-3, 200 ppm Sil@HIL, the PAO/JN-3 binary formulation and the PAO/JN-3/Sil@HIL ternary formulation. PIE values were compared to evaluate the additional improvement associated with the introduction of Sil@HIL into the PAO/JN-3 formulation and to compare the ternary formulation with the corresponding individual additives and binary formulation.

All additive systems inhibited wax deposition on the cold surface of the waxy model oil to different extents (Figure 7). When used individually, 40 ppm PAO, 100 ppm JN-3 and 200 ppm Sil@HIL yielded PIE values of 33.33%, 50.65% and 27.27%, respectively. JN-3 had the highest inhibition among the individual additives, whereas Sil@HIL had a relatively low PIE when used alone, indicating limited activity as a single additive in this experimental system.

Combining 40 ppm PAO with 100 ppm JN-3 increased PIE to 67.53%, 34.20 and 16.88 percentage points above the values for PAO and JN-3 alone, respectively. Adding 200 ppm Sil@HIL to this binary formulation further increased PIE to 77.49%, which is 9.96 percentage points above the PAO/JN-3 binary formulation and the highest value among the systems examined.

Singh et al. [39] similarly found that an EVA/SiO_2_ nanocomposite PPD improved low-temperature flowability more than EVA alone, indicating that combining nano-SiO_2_ with a polymer can strengthen the regulation of wax-crystal structure and low-temperature flow in waxy crude oil. This result is consistent with the further increase in PIE observed here when Sil@HIL was introduced into the PAO/JN-3 formulation.

Thus, under the present conditions, the PAO/JN-3 binary formulation outperformed the corresponding individual additives, and introducing Sil@HIL produced a further improvement over the binary formulation. The possible basis for this improvement is considered below using wax-crystal morphology and mechanistic analysis.

Across all wax deposition inhibition efficiencies measured, the formulation containing 40 ppm PAO, 100 ppm JN-3 and 200 ppm Sil@HIL gave the highest wax deposition inhibition within the experimental range examined and was therefore selected as the ternary formulation.

### 2.7. Wax-Crystal Morphology

Figure 8 shows polarized light micrographs of wax crystals formed under the same thermal history and cooling conditions. Polarized light microscopy exploits the optical anisotropy of wax crystals to visualize their precipitation, growth and aggregation and is therefore suitable for comparing changes in microscopic structure after treatment with different flow improvers. Perna et al. [40] similarly used crossed polarized light microscopy to study waxy crude oil treated with a polymeric PPD and found that the additive markedly altered the wax-crystal phase morphology and microstructure at low temperatures, supporting the use of wax-crystal morphology as microscopic evidence for PPD action.

The blank contained readily apparent needle-like, plate-like and rod-like wax crystals, some of which crossed and interconnected to form aggregated structures (Figure 8). Al-Shboul et al. [41] likewise observed overlapping small wax crystals that formed a relatively regular network in an untreated waxy model oil. The pronounced crystal growth, aggregation and interconnection observed in the blank are therefore broadly consistent with reported wax-crystal morphologies in untreated waxy oils.

After PAO was added, the basic needle-like and plate-like morphology remained evident, without pronounced restructuring relative to the blank. PAO therefore had a comparatively limited direct effect on wax-crystal growth morphology under the static polarized light microscopy conditions used here. The magnitude of the microscopic morphological change in the PAO system therefore need not correspond one-to-one with its wax deposition inhibition in the cold-finger test.

Adding JN-3 produced clearer changes in crystal morphology and intercrystalline connections. Long crystals and continuously interconnected structures became less common, suggesting that JN-3 affected wax-crystal growth and aggregation. In a study of a poly(octadecyl acrylate)-based PPD, Yang et al. [42] found that the polymer converted large plate-like structures in a model wax oil into more regular and compact crystals, thereby weakening the formation of a continuous wax-crystal network. The reduced intercrystalline connections observed with JN-3 provide a microscopic basis for its relatively high wax deposition inhibition when used alone.

Sil@HIL alone also altered the wax-crystal structure to some extent, although distinct crystals and locally aggregated regions remained, indicating limited control when it was used alone. Yan et al. [43] found that EVA/polystyrene-microsphere composite PPDs provided only limited improvement when the micro/nanoscale component was added alone but further altered wax-crystal aggregation when combined with a polymer.

The PAO/JN-3 binary formulation further reduced the total crystalline area and clearly altered the wax-crystal distribution relative to the individual additive systems. After Sil@HIL was added, large needle-like and plate-like crystals and continuous connections were least evident in the ternary formulation. The field of view was dominated by smaller, more dispersed crystals, and the tendency to form a large-scale continuous network was further weakened. Yan et al. [43] showed that incorporating a micro/nanoscale component capable of providing additional interfacial interactions into a polymeric PPD can further regulate polymer-wax-crystal interactions and produce greater control of crystal structure than the polymer alone.

Overall, the additives affected wax-crystal structure differently. PAO produced comparatively little direct change in the microscopic morphology of static wax crystals, whereas JN-3 and Sil@HIL altered wax-crystal growth and aggregation to different extents. When all three components were present, large wax crystals and continuously interconnected structures were reduced further. These morphological changes provide microscopic support for the improved performance of the ternary formulation.

### 2.8. Mechanistic Analysis of the PAO/JN-3/SiO_2_-IL Ternary Formulation

Under static cold-finger conditions, the temperature difference between the cold finger and the bulk oil phase causes wax components in the low-temperature region to become supersaturated and precipitate, followed by nucleation, growth, aggregation and accumulation on the cold surface. The performance and polarized light microscopy results indicate that PAO, JN-3 and Sil@HIL play different functional roles in the ternary formulation. Their combined effect is therefore more appropriately interpreted as functional complementarity among distinct regulatory stages than as the result of a single mechanism.

The system remained static and was not mechanically sheared during the experiment. Wax components therefore migrated and accumulated on the cold-finger surface mainly under temperature and concentration gradients. Once dispersed in the oil phase, PAO polymer chains may indirectly affect the continued accumulation of wax on the cold surface by altering the local physical properties, the resistance to wax diffusion and migration, and the state of the deposition interface [7]. The absence of pronounced restructuring of wax-crystal morphology after PAO addition suggests that its effect on deposition may operate primarily through regulation of wax migration and deposition rather than through direct changes in wax-crystal growth morphology.

JN-3 appears to act mainly on wax-crystal formation and aggregation. Polymeric PPDs can disrupt the regular arrangement of paraffin molecules through co-crystallization, adsorption onto crystal surfaces and steric hindrance. These effects alter the wax-crystal size, morphology and aggregation and thereby reduce the ability of the crystals to form a continuous network [44].

Sil@HIL introduces a functionalized nanoscale interface. The high specific surface area of nano-SiO_2_ can provide heterogeneous-nucleation and interfacial-interaction sites, whereas the surface C6 alkyl-imidazolium structure may regulate the compatibility among the particles, the hydrocarbon oil phase and the wax crystals. Previous studies have shown that organically functionalized SiO_2_ can participate in wax-crystal nucleation and growth and alter the final crystal morphology and mode of aggregation [45].

The PAO/JN-3/Sil@HIL ternary formulation can therefore be described as a functionally complementary system that combines regulation of the macroscopic deposition process, wax-crystal structure and nanoscale interfaces. PAO may primarily influence the migration and accumulation of wax components toward the cold surface, JN-3 may disrupt wax-crystal growth and intercrystalline connections, and Sil@HIL may further regulate wax-crystal nucleation and aggregation through a functionalized nanoscale interface. The distinct and complementary stages affected by the three components may enable wax deposition to be inhibited at both the macroscopic transport and microscopic wax-crystal-structure scales, producing a combined regulatory effect greater than that of a single mode of action.

## 3. Materials and Methods

### 3.1. Materials

Diesel was used as the base liquid phase and paraffin as the solid wax phase; both were purchased from Guanlin Chemical Technology Co., Ltd. (Guangzhou, China). The diesel was a commercial No. 0 grade diesel with a density of 0.835 g cm^−3^ at 20 °C and an initial boiling point above 180 °C. The paraffin was fully refined paraffin wax with a melting point of 58–60 °C, an average carbon number of C_24_–C_28_ and an oil content below 0.5 wt%. Poly(α-olefin) suspension (PAO, milky white viscous liquid, active content 30 wt%, MW. 8–12 × 10^6^ g/mol) and the pour point depressant JN-3 (transparent dark yellow liquid, active content 42 wt%, MW. ~2 × 10^4^ g/mol) were purchased from Jiahua Technology Co., Ltd. (Jingzhou, China). Ethylene–vinyl acetate copolymer (EVA) was purchased from Macklin Inc. (Shanghai, China) and dissolved with the aid of xylene before use. Tetraethyl orthosilicate, imidazole, 3-chloropropyltrimethoxysilane (CPTMS), chloroethane, chlorobutane and chlorohexane were purchased from Macklin Inc. (Shanghai, China). Triethylamine, aqueous ammonia, xylene and the other reagents were of analytical grade. All ppm values reported here refer to the mass of the active additive component relative to the total mass of the model oil.

### 3.2. Preparation of Samples and Formulations

#### 3.2.1. Preparation of Waxy Model Oil

Paraffin and diesel were weighed according to the paraffin mass fraction specified for each experiment. The paraffin was added to the diesel, and the mixture was heated to 60 °C under continuous stirring until the paraffin dissolved completely, giving a homogeneous waxy model oil. Model oils with different wax contents were prepared by adjusting the amounts of paraffin and diesel while keeping the total sample mass constant.

#### 3.2.2. Preparation of PAO/PPD Binary Formulations

PAO/PPD binary formulations were prepared by physical blending. The quantities of PAO drag-reducing agent and PPD required to reach the specified concentrations were added separately to the pre-prepared waxy model oil. The formulation was then stirred at 60 °C for 20 min to disperse both additives fully in the oil phase, to ensure complete dissolution of the paraffin and to standardize the thermal history of all samples. After treatment, the samples were cooled to the specified experimental temperature using the same cooling procedure and were then used to evaluate wax deposition performance [46].

#### 3.2.3. Preparation of SiO_2_ and SiO_2_-IL

Nano-SiO_2_ was first prepared by a sol–gel method. Absolute ethanol (80.0 mL) and concentrated aqueous ammonia (5.0 mL) were added sequentially to a 250 mL three-necked flask and mixed by magnetic stirring for 5 min. Deionized water (5 mL) was then added slowly, and stirring was continued for 5 min to obtain a homogeneous ammonia/ethanol/water mixture. Finally, tetraethyl orthosilicate (10.0 mL) was added dropwise to this solution under continuous stirring. After reacting at room temperature for 24 h, the product was centrifuged at 4000 r/min for 20 min in a benchtop centrifuge, washed alternately with absolute ethanol and deionized water, and dried to obtain nano-SiO_2_ particles.

Nano-SiO_2_ particles (30.0 g), CPTMS (40.0 mL) and xylene (80.0 mL) were then mixed thoroughly in a flask and heated to 100 °C. After 12 h, chlorinated nano-SiO_2_ particles (Sil@Cl) were obtained. Sil@Cl (30.0 g), imidazole (25.0 g), triethylamine (20.0 mL) and xylene (80 mL) were mixed in a flask and heated to 100 °C. After 8 h, imidazole-modified silica (Sil@imidazole) was obtained. Sil@imidazole (8.0 g) and xylene (80.0 mL) were placed in each of three flasks, after which chloroethane, chlorobutane or chlorohexane (10.0 mL) was added to each flask. Heating at 100 °C for 12 h yielded three IL-modified silica materials, designated Sil@EIL, Sil@BIL and Sil@HIL, respectively.

#### 3.2.4. Preparation of the PAO/PPD/SiO_2_-IL Ternary Formulation

The specified quantities of PAO drag-reducing agent, PPD and SiO_2_-IL were added sequentially to the pre-prepared waxy model oil to reach the target concentration of each component. The formulation was maintained at 60 °C throughout blending. PAO and PPD were added first and stirred until they were fully dispersed in the oil phase. SiO_2_-IL was then added slowly, and the formulation was stirred for a further 20 min to promote uniform dispersion of SiO_2_-IL in the oil phase. To ensure comparability among experimental groups, all samples were prepared with the same order of addition, mixing temperature, stirring time and thermal-history treatment. After blending, the samples were brought to the specified experimental temperature using the same temperature-control procedure and then evaluated in subsequent cold-finger wax deposition tests.

### 3.3. Material Characterization

#### 3.3.1. Fourier Transform Infrared Spectroscopy (FT-IR)

Infrared spectra were obtained with a Nicolet iS20 Fourier transform infrared spectrometer (Thermo Fisher Scientific Inc., Waltham, MA, USA). Samples were prepared as KBr pellets and measured over 4000–500 cm^−1^ at a resolution of 4 cm^−1^ with 32 scans. Characteristic functional groups and chemical structures were identified from the positions and relative intensities of the absorption bands.

#### 3.3.2. Thermogravimetric Analysis (TGA)

Thermogravimetric analysis was performed using a Discovery TGA 55 (TA Instruments, New Castle, DE, USA). Measurements were conducted under a high-purity nitrogen atmosphere at a flow rate of 50 mL min^−1^. The samples were heated from room temperature to 800 °C at 10 °C min^−1^. Thermal stability and decomposition behavior were evaluated from the variation in sample mass with temperature.

#### 3.3.3. Scanning Electron Microscopy (SEM)

The microscopic morphologies of unmodified SiO_2_ and the SiO_2_-IL samples were characterized by MIRA3 scanning microscope (TESCAN, Brno, Czech Republic). Before imaging, the dried powder samples were sputter-coated with gold. An accelerating voltage of 5 kV was used. Images acquired at different magnifications were used to examine particle morphology, size, surface structure and aggregation.

### 3.4. Performance Evaluation

#### 3.4.1. Wax Deposition Apparatus and Evaluation Method

Wax deposition is commonly evaluated using a cold-finger test. A cold-finger apparatus is structurally simple and compact, easy to operate, capable of precise temperature control and requires only a small oil volume [47]. By establishing a stable temperature difference between the cold-finger surface and a waxy oil sample, the apparatus simulates wax deposition on a low-temperature wall. It is therefore widely used to investigate wax deposition in waxy crude oils and their emulsions and to evaluate wax inhibitors [48].

The cold-finger wax deposition apparatus comprised a thermostatic hot bath, a low-temperature cold bath, a cold finger and a sample beaker. The sample beaker was placed in the thermostatic hot bath, whose circulating heat-transfer medium maintained the bulk temperature of the waxy model oil at the specified value. The cold bath was connected to the cold finger and circulated a coolant to control the cold-finger temperature and maintain its surface at the specified low temperature. During the experiment, the cold-finger surface was colder than the bulk waxy model oil, producing a stable temperature gradient between the cold-finger surface and the bulk oil phase that promoted the migration, precipitation and deposition of wax components on the cold surface. All deposition experiments were carried out under static conditions: the model oil was not stirred, and no flow was imposed during deposition, so that the measurements characterize wax deposition driven by the temperature and concentration gradients alone.

Before each experiment, the prepared waxy model oil was heated and stirred thoroughly at 60 °C to dissolve the paraffin completely, to disperse the added PAO drag-reducing agent, PPD and SiO_2_-IL, and to standardize the thermal history of all samples. A specified amount of treated model oil was then transferred to a beaker and placed in the thermostatic hot bath. The oil temperature was adjusted to 55 °C and allowed to stabilize. The cold bath was then switched on, the cold finger was adjusted to 25 °C, and the finger was immersed vertically to a fixed position in the waxy model oil. The system remained static during deposition, without mechanical stirring. Timing began once the temperatures of the bulk oil phase and cold-finger surface were stable. At the specified deposition time, the cold finger was removed slowly and adhering liquid oil was allowed to drain under standardized conditions. Three parallel experiments were performed for each condition, and results are reported as the mean ± standard deviation.

The results were calculated as follows:

*m_d_* = *m*_1_ − *m*_2_ (1)

where *m_d_* is the mass of wax deposit, *m*_1_ is the total mass of the sample beaker and oil before deposition, and *m*_2_ is the total mass of the sample beaker and oil after deposition, in g.

(2) $$PIE=\frac{W_{f}-W_{t}}{W_{f}}\times 100\%$$

where *W_f_* is the mean mass of wax deposit in the blank sample, *W_t_* is the mean mass of wax deposit in the corresponding additive-containing sample, and PIE is the wax deposition inhibition efficiency [49,50], in %.

(3) $$SD=\sqrt{\frac{\sum_{i=1}^{n}{\left(x_{i}-\bar{x}\right)}^{2}}{n-1}}$$

where *x_i_* is the result of an individual experiment, *x* is the mean of the group, *n* is the number of experiments and *SD* is the sample standard deviation.

#### 3.4.2. Observation of Wax-Crystal Morphology

A polarized light microscope equipped with a temperature-controlled cold stage was used to examine wax-crystal morphology in model oil containing 20 wt% paraffin. A small amount of the sample was placed on a microscope slide and covered with a coverslip. The sample was held at 60 °C for 30 min to eliminate its thermal history, cooled to 25 °C at 1 °C min^−1^ and held for 10 min before polarized light micrographs were acquired at ×200 magnification.

### 3.5. Experimental Design

Previous studies have shown that the performance of PAO drag-reducing agents is strongly dependent on polymer concentration, flow conditions and mechanical degradation. Chen et al. [51] investigated PAO at concentrations of 5–200 ppm and included 40 ppm in their mechanical-degradation experiments. The effect of PAO on wax deposition is likewise concentration-dependent. Lv et al. [7] reported that a low dosage of drag-reducing agent produced only a limited reduction in wax deposition, whereas higher dosages could increase wax deposition under certain conditions because of enhanced polymer adhesion. These findings indicate that increasing PAO concentration does not necessarily lead to a continuous improvement in wax deposition inhibition. Considering the concentration range examined in previous studies and the need to maintain a relatively low PAO dosage in the present formulation study, 40 ppm was adopted as the fixed PAO concentration for the subsequent wax-deposition and formulation experiments.

#### 3.5.1. Determination of the Paraffin Content of the Model Oil

Paraffin content is an important compositional parameter governing the low-temperature flow and wax-precipitation behavior of waxy model oils. Helsper and Liberatore [52] investigated wax precipitation in model oils containing 5, 10, 15 and 20 wt% paraffin and found that paraffin content markedly affected the wax appearance temperature. Under otherwise identical conditions, increasing the paraffin content from 5 wt% to 20 wt% raised the wax appearance temperature by as much as about 13 °C, indicating that paraffin content is an important determinant of wax-phase formation in model oils.

A paraffin content of 20 wt% has also been used as a representative composition in previous studies of high-wax-content model oils. When investigating the combined effects of modified EVA and asphaltenes on the flowability of waxy oil, Liu et al. [53] dissolved paraffin in No. 0 diesel to prepare a model oil containing 20 wt% paraffin and used this homogeneous model oil to evaluate different additive systems. That diesel-paraffin composition was similar to the model oil used here.

Paraffin mass fraction was the sole variable in this experiment and was set at five levels: 10, 15, 20, 25 or 30 wt%. The total sample mass was fixed at 100 g, the PAO dosage at 40 ppm and, with reference to previous cold-finger studies, the deposition time at 2 h for this stage [49]. All other cold-finger conditions were held constant. The mass of wax deposit and the reproducibility of the parallel experiments at each paraffin content were compared to identify a model-oil composition that provided a measurable deposited mass, straightforward operation and good experimental reproducibility.

#### 3.5.2. Determination of a Standardized Deposition Time

Deposition time is an important experimental parameter affecting the mass of wax deposited on the cold finger. Previous studies have shown that wax deposition generally increases rapidly at first and then gradually slows. If the deposition time is too short, the deposited mass may be too small to weigh accurately or to distinguish among additive formulations. Excessively long experiments increase the testing period and may allow factors such as deposit aging to affect the results.

Lee et al. [28] found that wax deposition on a cold surface was strongly time-dependent, with the deposit growing over time and gradually approaching a stable state. Lim et al. [49] used a cold-finger method to screen different surfactants and surfactant/SiO_2_-nanoparticle formulations, with a standardized experimental time of 2 h while comparing the deposited mass and inhibition performance of different additive systems under identical conditions. Their design provides an experimental basis for using a fixed deposition time in the comparative evaluation of different wax-inhibition systems.

Accordingly, with the PAO dosage fixed at 40 ppm, deposition time was the sole variable and was set at six time points: 0.5, 1, 2, 3, 4 or 6 h. Three parallel experiments were conducted under each condition. The total sample mass was fixed at 100 g, the paraffin content was set to the value selected in the preceding experiment, and all other cold-finger conditions were held constant. The mass of wax deposit, experimental reproducibility and testing efficiency at the different times were compared to determine a common evaluation time for subsequent tests of individual additives and formulations.

Previous work indicates that the effects of PAO drag-reducing agents on wax deposition depend strongly on concentration and hydrodynamic conditions [7]. In flowing systems, PAO can influence heat and mass transfer, whereas its direct regulation of wax-crystal nucleation, growth and aggregation is limited. Under the static cold-finger conditions used here, the contribution of PAO was therefore considered mainly in terms of its possible influence on wax migration and accumulation near the cold surface. Because wax deposition is also governed by wax precipitation, aggregation and gel-network formation [54], a PPD capable of directly modifying wax-crystal structure was introduced to construct a functionally complementary PAO/PPD formulation.

#### 3.5.3. Performance Evaluation Methods of PAO/PPD Binary Formulations

Previous studies have shown that combining wax deposition control strategies with different modes of action can further improve wax inhibition. Wang et al. [55] examined the combined effect of a magnetic field and a ferromagnetic nanocomposite PPD (FNPPD) on wax deposition. The combined treatment inhibited deposition more effectively than either treatment alone, yielding a wax deposition inhibition efficiency of 30.11%, which exceeded the sum of the effects of the two individual treatments. This finding indicates that complementary regulatory mechanisms can strengthen wax deposition control.

Under the static cold-finger conditions used in this study, PAO may influence wax deposition by altering local physical properties and the migration and accumulation of wax components near the cold surface, whereas EVA and JN-3 primarily regulate wax-crystal nucleation, growth and aggregation. We therefore fixed the PAO dosage at 40 ppm and combined it with EVA or JN-3 at 20, 40, 60, 80 or 100 ppm to form binary formulations. Each sample had a total mass of 100 g. The paraffin mass fraction and deposition time were fixed at the values selected in the preceding experiments; all other cold-finger conditions were held constant, and three parallel experiments were performed for each condition. Mean deposited mass and wax deposition inhibition efficiency (PIE) were compared among the formulations containing different PPDs at different concentrations. These comparisons were used to evaluate the effects of EVA and JN-3 dosage on the wax deposition performance of the PAO/PPD binary formulations and to select an appropriate concentration of each PPD for combination with 40 ppm PAO.

After suitable concentrations of EVA and JN-3 had been selected, a blank, 40 ppm PAO alone, EVA alone, JN-3 alone, the PAO/EVA binary formulation and the PAO/JN-3 binary formulation were tested. The concentrations of EVA and JN-3 in the single-additive groups were identical to those in the corresponding formulations, and all other experimental conditions were held constant. Mean deposited mass and PIE were compared among the formulations to evaluate the wax deposition inhibition achieved by combining PAO with each PPD and the improvement relative to the respective individual additives. These results were also used to select the PAO/PPD base formulation for the subsequent ternary-formulation experiments.

#### 3.5.4. Screening of SiO_2_-IL Alkyl-Chain Length and Dosage

After screening the PAO/PPD binary formulations, SiO_2_-IL was introduced into the selected binary formulation to construct a PAO/PPD/SiO_2_-IL ternary formulation and further improve wax deposition inhibition.

Because the alkyl-chain structure of the IL on the SiO_2_-IL surface and the SiO_2_-IL dosage may both affect particle dispersion in the oil phase, interfacial interactions and interactions with wax components, the ternary formulations were screened systematically over combinations of chain length and dosage. Three SiO_2_-IL materials with C2, C4 or C6 alkyl chains, namely Sil@EIL, Sil@BIL and Sil@HIL, were tested at 0, 50, 100, 200, 300 or 500 ppm. The 0 ppm group was the PAO/PPD binary formulation without SiO_2_-IL and served as the common control.

The total sample mass was fixed at 100 g. The PAO and PPD dosages, paraffin mass fraction and deposition time were fixed at the values selected in the preceding experiments. All other cold-finger conditions were held constant, and three parallel experiments were performed for each condition. Mean deposited mass and wax deposition inhibition efficiency were compared among the different SiO_2_-IL chain lengths and dosages. These comparisons were used to evaluate the effects of the surface alkyl-chain structure and dosage of SiO_2_-IL on the wax deposition performance of the PAO/PPD base formulation and to select the SiO_2_-IL structure and dosage for subsequent ternary-formulation experiments.

#### 3.5.5. Wax Deposition Inhibition Performance of the PAO/PPD/SiO_2_-IL Ternary Formulation

After screening the PAO/PPD binary formulation and the SiO_2_-IL alkyl-chain length and dosage, results obtained under the same standardized cold-finger conditions were compiled for comparative evaluation. Six systems were included: the blank, PAO alone, PPD alone, SiO_2_-IL alone, the PAO/PPD binary formulation and the selected PAO/PPD/SiO_2_-IL ternary formulation. PAO and PPD were used at the concentrations selected in the preceding experiments, whereas SiO_2_-IL was used at the selected alkyl-chain structure and dosage. Previously obtained results for systems evaluated under identical conditions were used directly in this comparison, while the SiO_2_-IL-alone system was independently evaluated under the same experimental conditions.

Wax deposition inhibition efficiency (PIE) was compared among the individual additives, binary formulation and ternary formulation. Attention was given to the difference between the PAO/PPD binary formulation and the corresponding ternary formulation to determine whether the incorporation of SiO_2_-IL provided an additional improvement in wax deposition inhibition.

## 4. Conclusions

We constructed a PAO/JN-3/SiO_2_-IL ternary formulation and evaluated its control of wax deposition in a high-wax-content model oil using a cold-finger method. The main conclusions are as follows:

(1) FT-IR, TGA and SEM indicated that alkyl-imidazolium organic structures were successfully introduced onto the SiO_2_ surface without disrupting the basic particle morphology, thereby providing a material basis for incorporating SiO_2_-IL into the subsequent formulations.
(2) Under the preliminary screening conditions with 40 ppm PAO, the deposited mass generally increased with paraffin content, and the model oil containing 20 wt% paraffin provided good measurement discrimination and reproducibility. The deposited mass increased as the deposition time was extended from 0.5 to 2 h and then remained at a similar level overall. Therefore, 20 wt% paraffin and a deposition time of 2 h were selected as the common evaluation conditions for subsequent formulation experiments.
(3) Combining PAO with a PPD further increased wax deposition inhibition. Within the JN-3 range of 20–100 ppm examined, formulation performance increased with dosage, and the binary formulation containing 40 ppm PAO and 100 ppm JN-3 achieved a wax deposition inhibition efficiency of 67.53%. Sil@HIL (C6) showed the highest performance. Its concentration response first increased and then decreased, with the highest wax deposition inhibition obtained at 200 ppm.
(4) The ternary formulation containing 40 ppm PAO, 100 ppm JN-3 and 200 ppm Sil@HIL achieved a wax deposition inhibition efficiency of 77.49%, 9.96 percentage points above that of the PAO/JN-3 binary formulation. Taken together, the performance and microscopic morphology suggest that the improvement may be associated with complementary effects among the PAO polymer, regulation of wax-crystal growth and aggregation by JN-3, and the functionalized nanoscale interfaces provided by Sil@HIL. The specific pathways require further experimental verification.

## Figures and Tables

**Figure 1:**
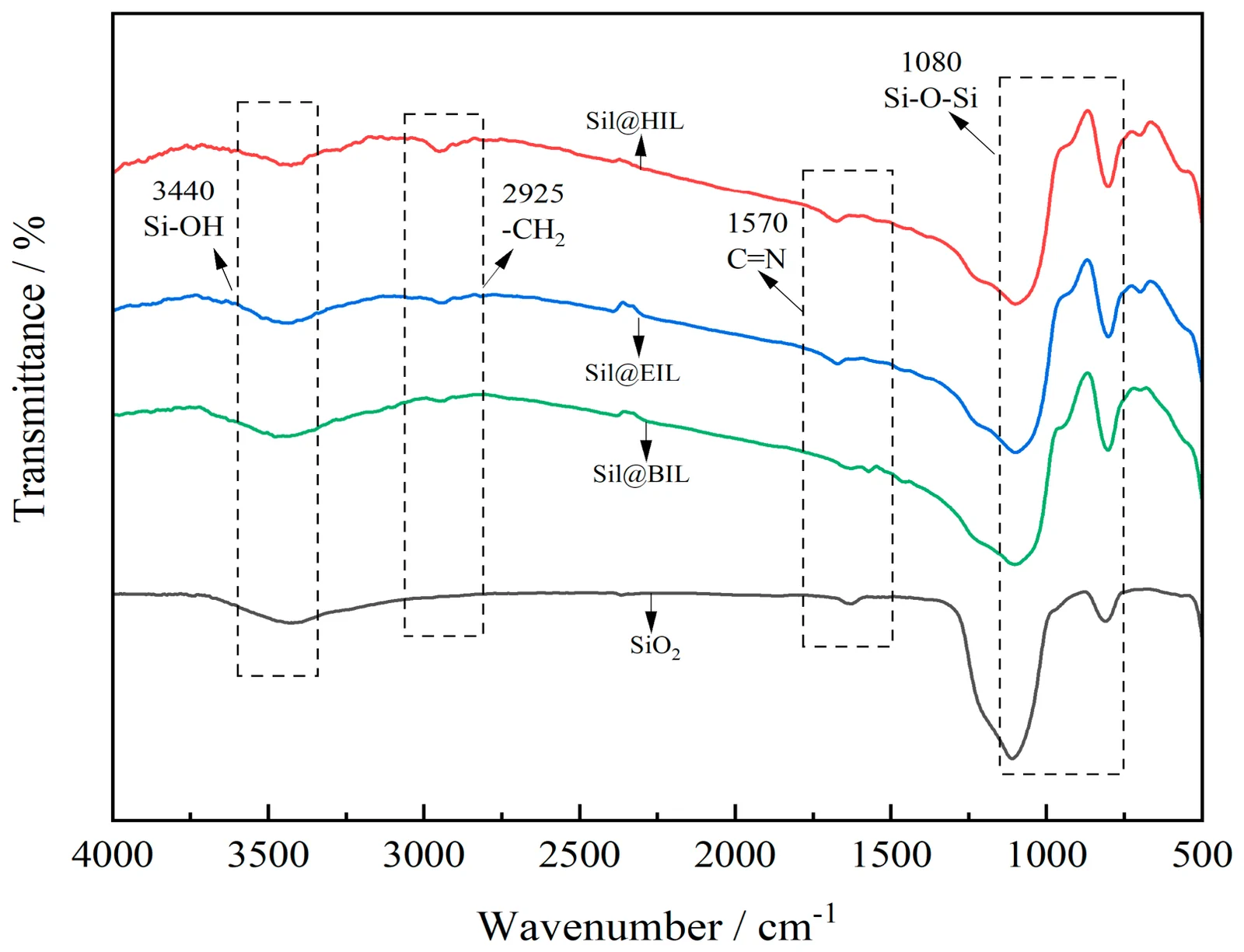
FT-IR spectra of SiO_2_ and SiO_2_-IL samples modified with ILs of different alkyl-chain lengths.

**Figure 2:**
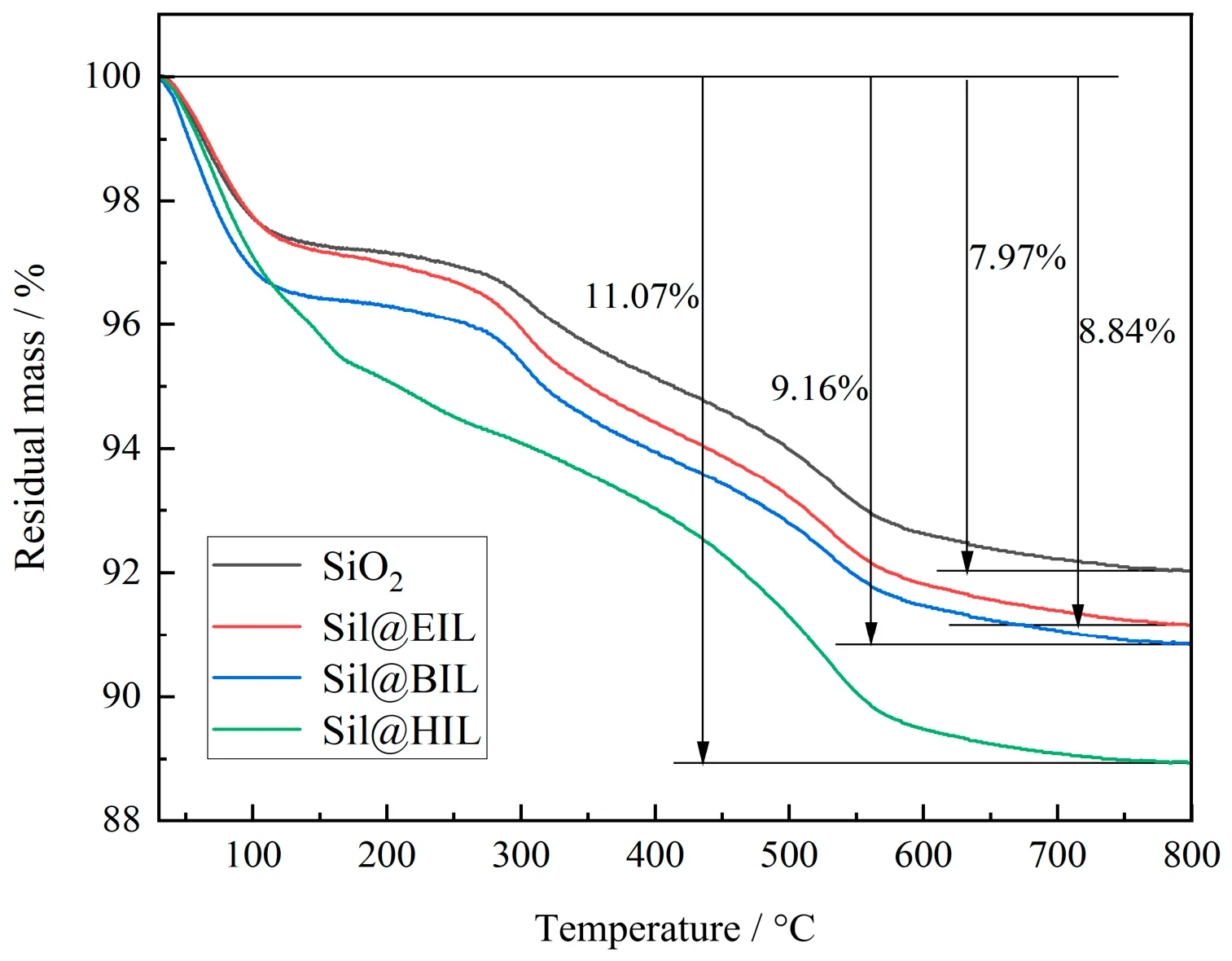
Thermogravimetric curves of SiO_2_ and SiO_2_-IL samples modified with ILs of different alkyl-chain lengths.

**Figure 3:**
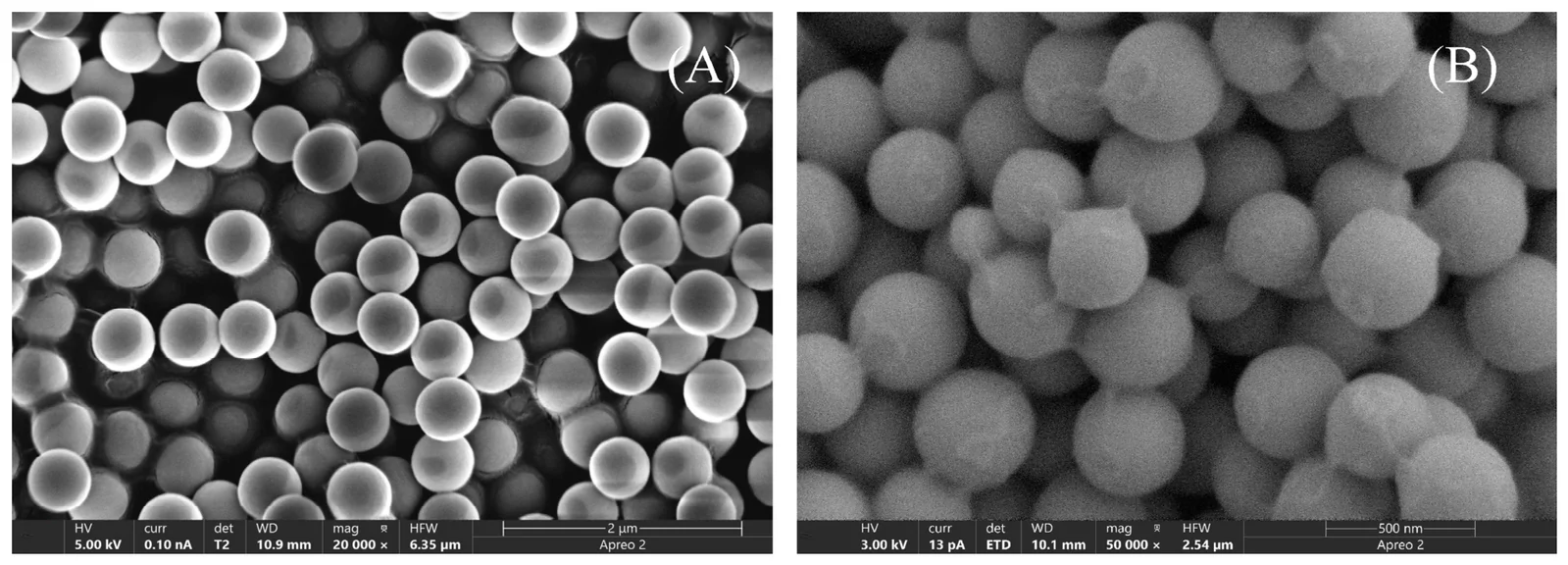
SEM images of (**A**) unmodified nano-SiO_2_ and (**B**) Sil@HIL.

**Figure 4:**
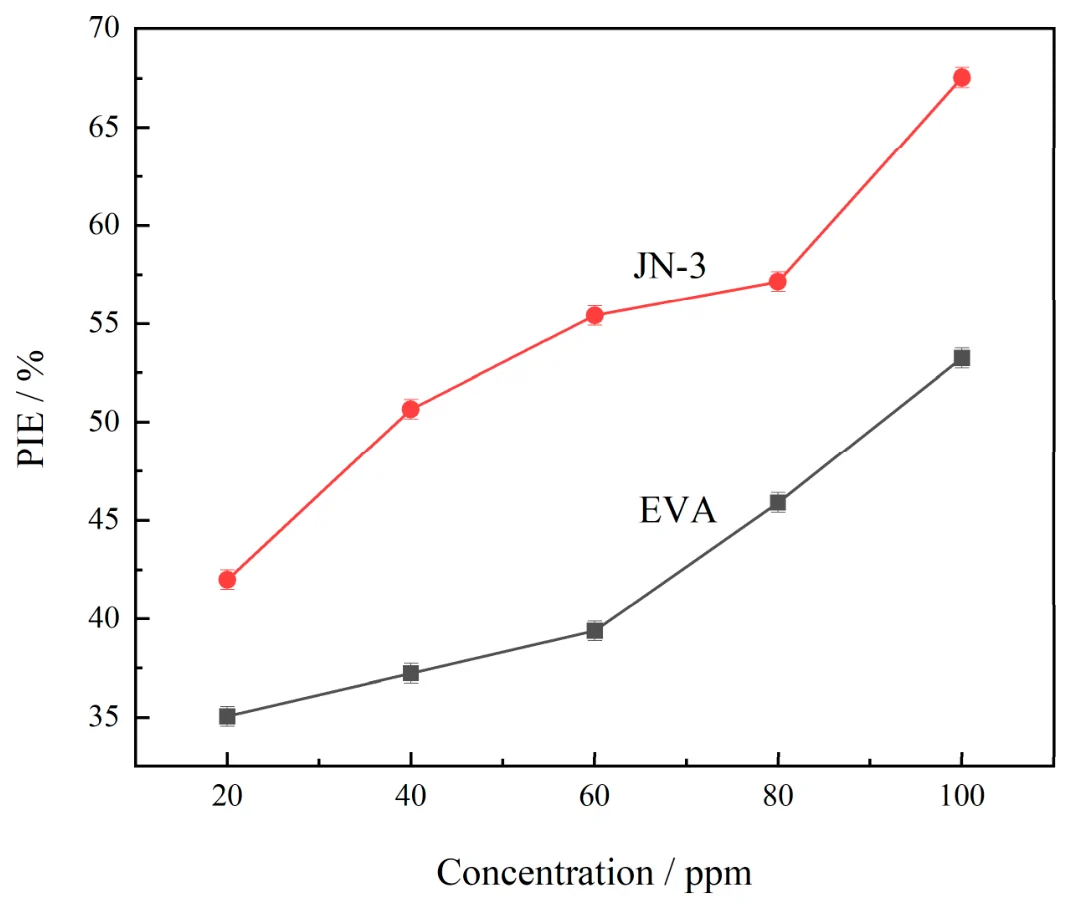
Wax deposition inhibition efficiency of 40 ppm PAO combined with different concentrations of EVA or JN-3. Error bars represent the standard deviations of three independent replicates.

**Figure 5:**
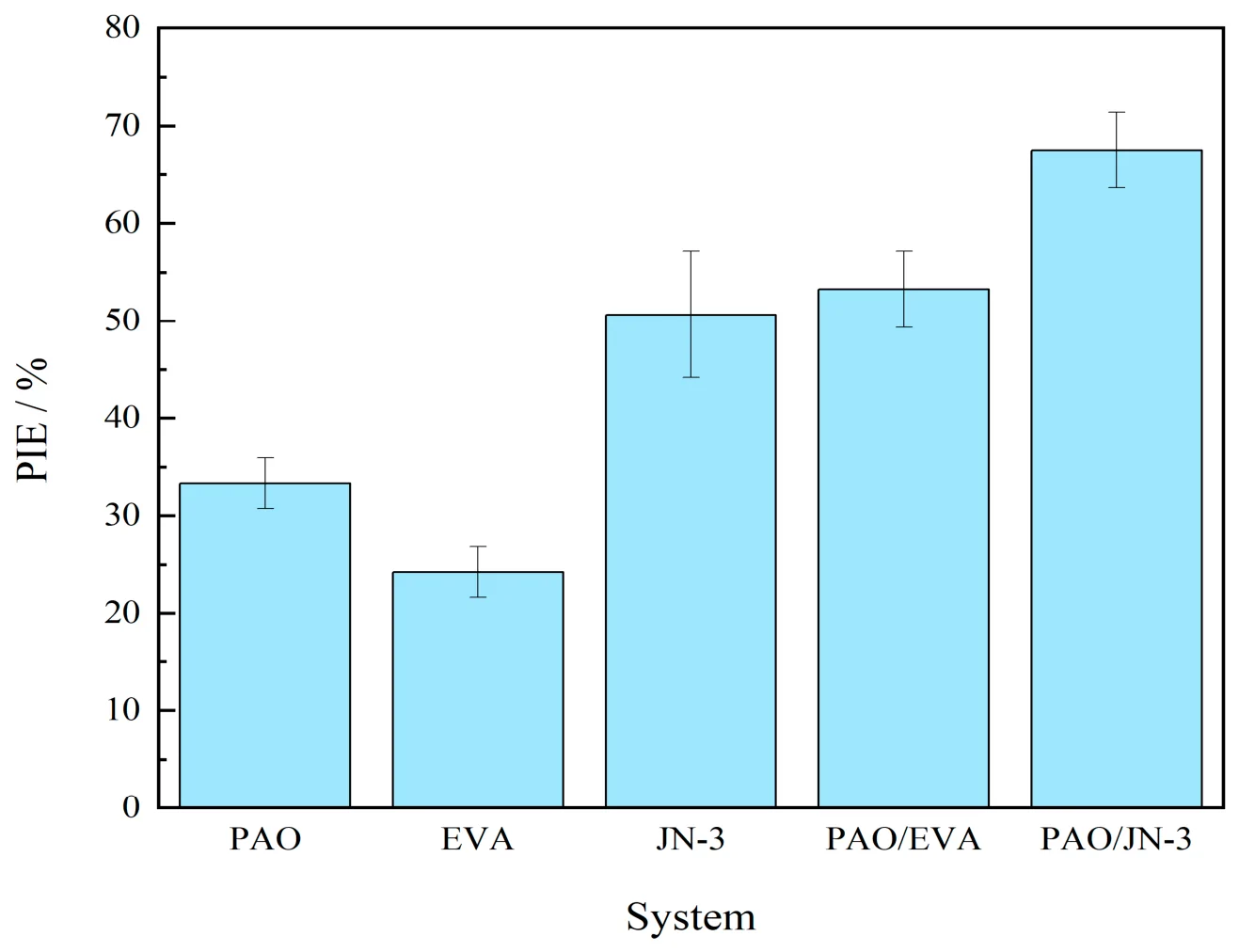
Comparison of wax deposition inhibition efficiencies for individual additives and binary formulations. Error bars represent the standard deviations of three independent replicates.

**Figure 6:**
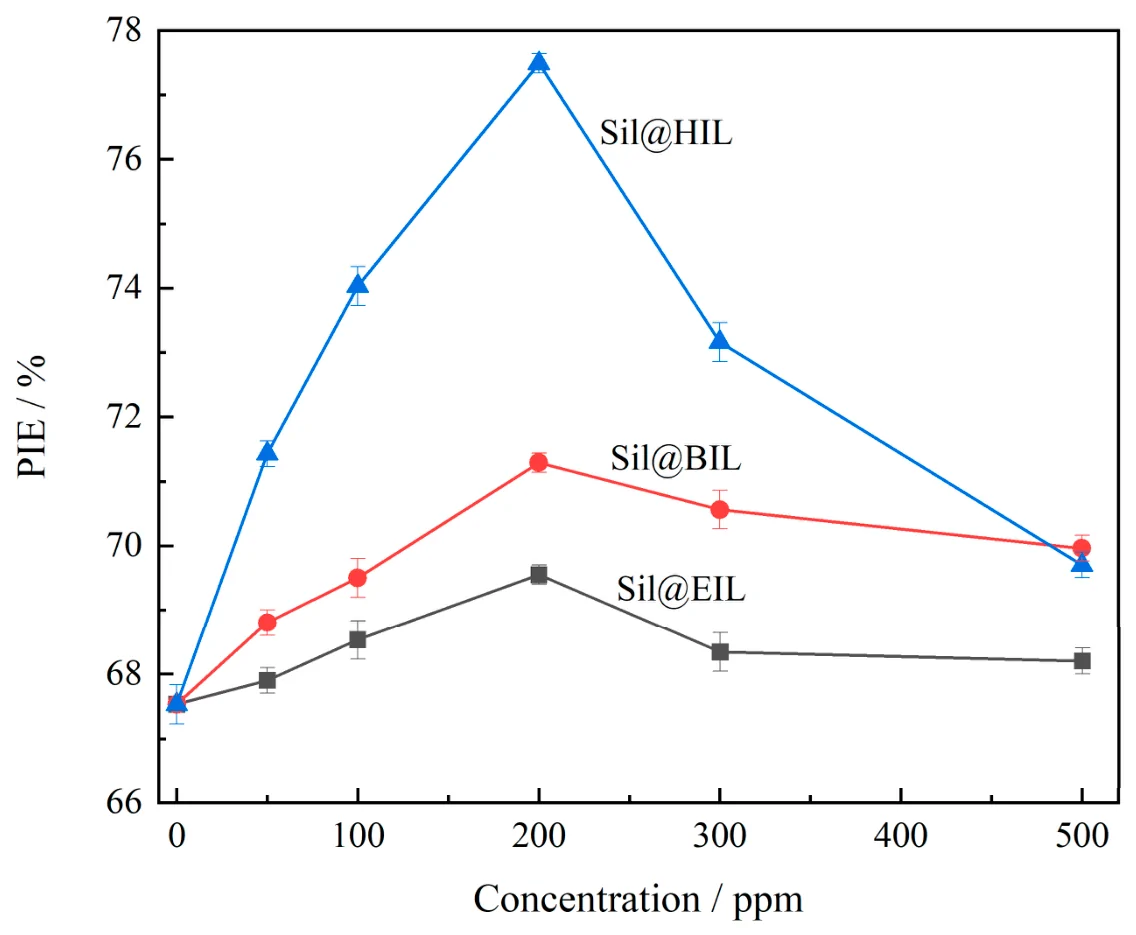
Effects of SiO_2_-IL alkyl-chain length and dosage on wax deposition inhibition by the PAO/JN-3 formulation. Error bars represent the standard deviations of three independent replicates.

**Figure 7:**
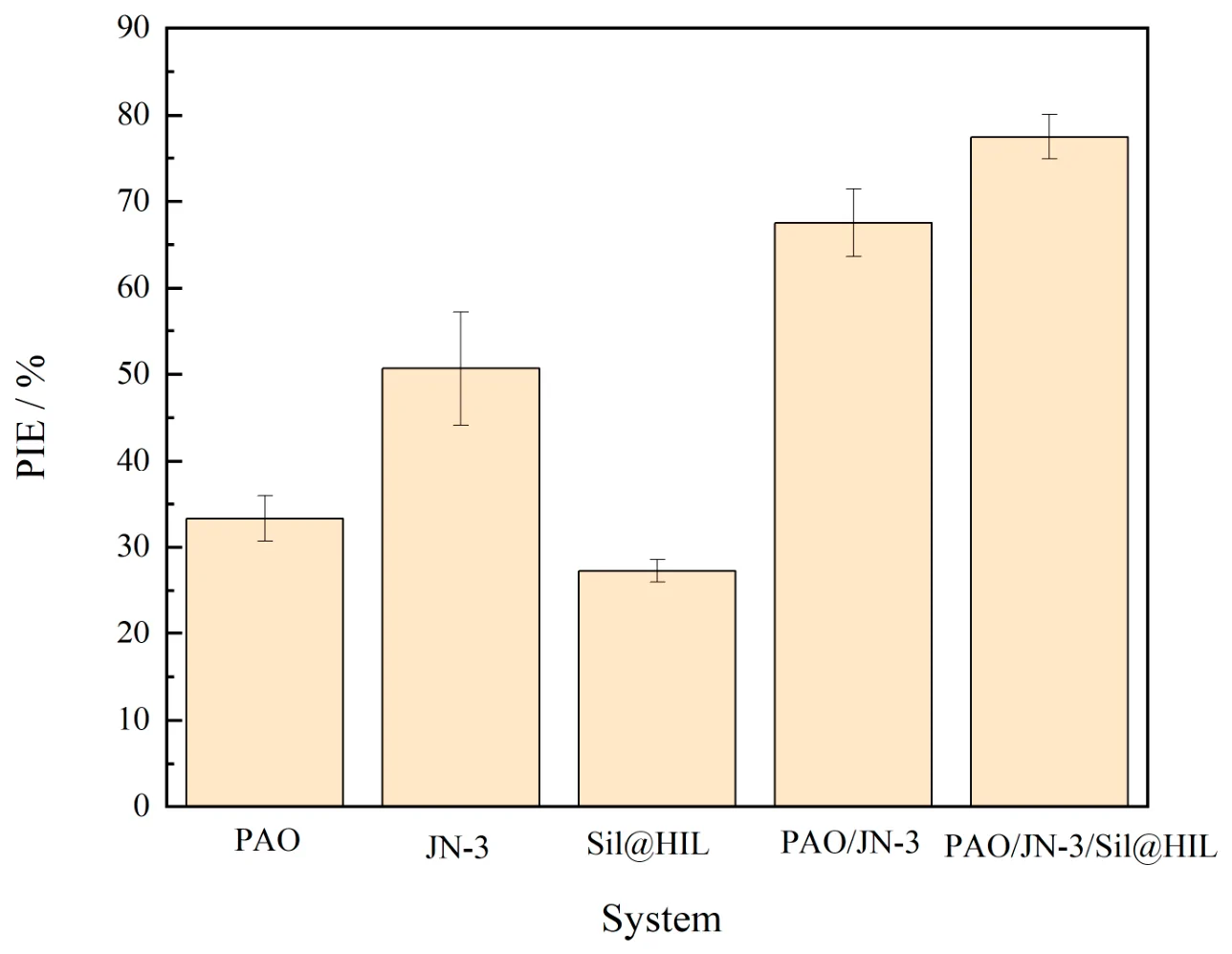
Comparison of wax deposition inhibition efficiencies for individual additives, the binary formulation and the ternary formulation. Error bars represent the standard deviations of three independent replicates.

**Figure 8:**
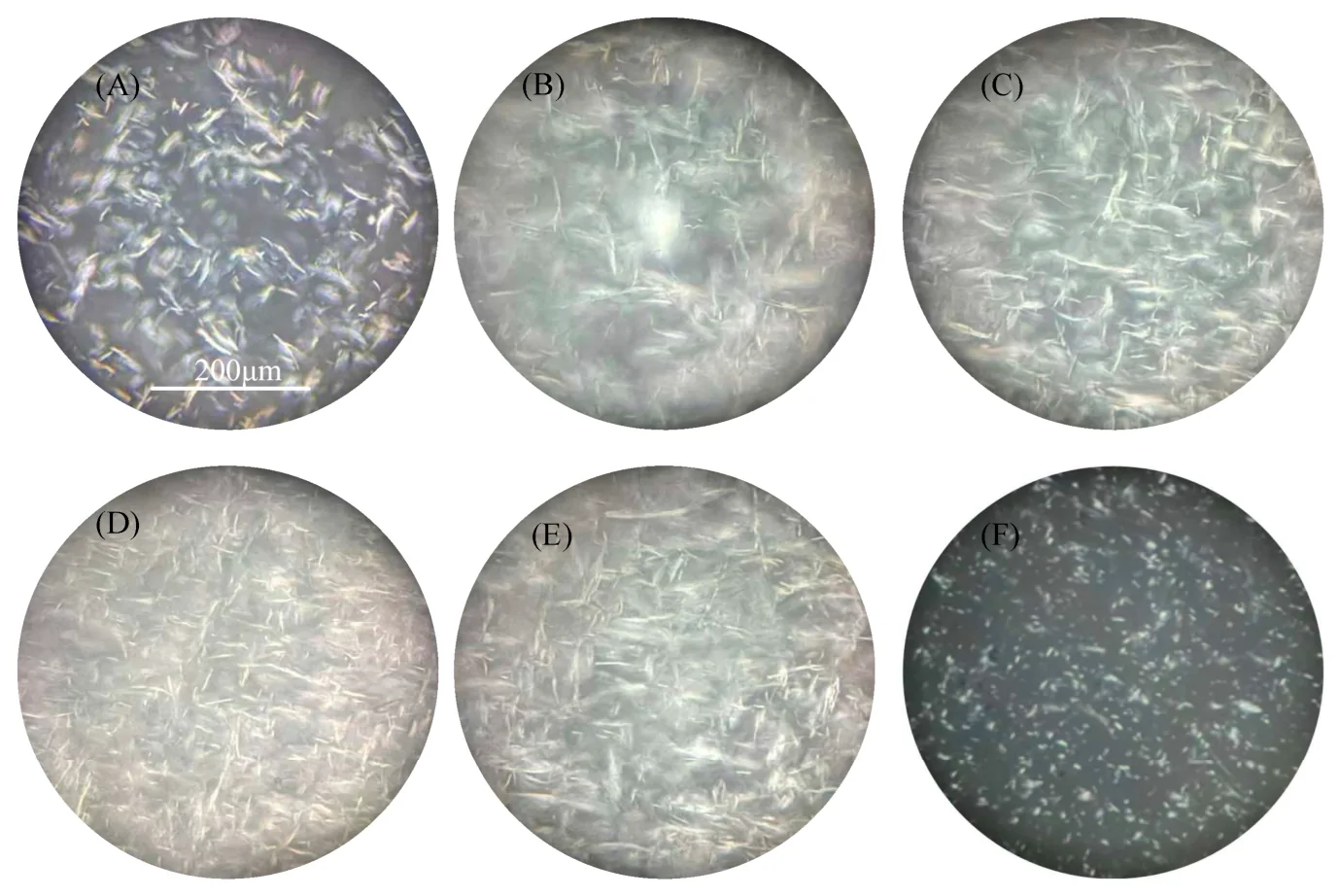
Polarized light micrographs of wax crystals in different additive systems: (**A**) blank; (**B**) 40 ppm PAO; (**C**) 100 ppm JN-3; (**D**) 200 ppm Sil@HIL; (**E**) 40 ppm PAO/100 ppm JN-3; and (**F**) 40 ppm PAO/100 ppm JN-3/200 ppm Sil@HIL.

**Table 1:** Results of paraffin content on wax deposition.

Paraffin Content /(wt%)	Mass Before Deposition $m_{1}$/(g)	Mass After Deposition $m_{2}$/(g)	Deposited Mass $m_{d}$/(g)	Mean Deposited Mass/(g)
10	158.20	158.00	0.20	0.19 ± 0.01
	157.88	157.70	0.18	
	158.39	158.20	0.19	
15	158.60	158.30	0.30	0.32 ± 0.03
	158.30	158.00	0.30	
	158.45	158.10	0.35	
20	157.45	156.93	0.52	0.51 ± 0.02
	157.84	157.35	0.49	
	158.56	158.03	0.53	
25	158.85	157.90	0.95	1.02 ± 0.07
	158.95	157.93	1.02	
	158.88	157.80	1.08	
30	159.25	157.60	1.65	1.95 ± 0.28
	159.00	157.01	1.99	
	159.06	156.85	2.21	

**Table 2:** Results of deposition time on wax deposition.

Deposition Time (h)	Mass Before Deposition $m_{1}$ (g)	Mass After Deposition $m_{2}$ (g)	Deposited Mass $m_{d}$ (g)	Mean Deposited Mass (g)
0.5	158.24	158.01	0.23	0.25 ± 0.02
	157.94	157.69	0.25	
	158.41	158.15	0.26	
1	158.18	157.80	0.38	0.40 ± 0.02
	158.64	158.22	0.42	
	157.82	157.43	0.39	
2	157.45	156.93	0.52	0.51 ± 0.02
	157.84	157.35	0.49	
	158.56	158.03	0.53	
3	158.00	157.52	0.48	0.48 ± 0.01
	158.38	157.90	0.48	
	157.93	157.46	0.47	
4	158.74	158.24	0.50	0.51 ± 0.02
	158.42	157.89	0.53	
	158.10	157.60	0.50	
6	158.89	158.41	0.48	0.48 ± 0.01
	158.55	158.07	0.48	
	158.34	157.85	0.49	

**Table 3:** Effect of combining different EVA concentrations with 40 ppm PAO on wax deposition.

EVA Concentration (ppm)	Mass Before Deposition $m_{1}$ (g)	Mass After Deposition $m_{2}$ (g)	Deposited Mass $m_{d}$ (g)	Mean Deposited Mass (g)
20	158.97	158.48	0.49	0.50 ± 0.01
	158.88	158.37	0.51	
	159.05	158.55	0.50	
40	158.86	158.38	0.48	0.48 ± 0.01
	158.78	158.30	0.48	
	158.72	158.23	0.49	
60	158.68	158.20	0.48	0.47 ± 0.02
	158.59	158.15	0.44	
	158.90	158.42	0.48	
80	158.68	158.25	0.43	0.42 ± 0.02
	158.66	158.24	0.42	
	158.60	158.20	0.40	
100	158.67	158.28	0.39	0.36 ± 0.03
	158.85	158.50	0.35	
	158.77	158.43	0.34	

**Table 4:** Effect of combining different JN-3 concentrations with 40 ppm PAO on wax deposition.

JN-3 Concentration (ppm)	Mass Before Deposition $m_{1}$ (g)	Mass After Deposition $m_{2}$ (g)	Deposited Mass $m_{d}$ (g)	Mean Deposited Mass (g)
20	158.86	158.41	0.45	0.45 ± 0.03
	158.79	158.37	0.42	
	158.98	158.51	0.47	
40	158.76	158.37	0.39	0.38 ± 0.01
	158.84	158.46	0.38	
	158.67	158.3	0.37	
60	158.82	158.46	0.36	0.34 ± 0.02
	158.63	158.28	0.35	
	158.75	158.43	0.32	
80	158.64	158.3	0.34	0.33 ± 0.03
	158.52	158.22	0.3	
	158.59	158.24	0.35	
100	158.88	158.59	0.29	0.25 ± 0.03
	158.71	158.48	0.23	
	158.6	158.37	0.23	

**Table 5:** Wax deposition performance of individual additives and binary formulations.

System	Mean Deposited Mass (g)	PIE (%)
blank	0.77 ± 0.04	0
40 ppm PAO	0.51 ± 0.02	33.33
100 ppm EVA	0.58 ± 0.02	24.24
100 ppm JN-3	0.38 ± 0.05	50.65
40 ppm PAO/100 ppm EVA	0.36 ± 0.03	53.25
40 ppm PAO/100 ppm JN-3	0.25 ± 0.03	67.53

## Data Availability

Data are contained within the article.
